# The UDP-glucuronosyltransferases of the blood-brain barrier: their role in drug metabolism and detoxication

**DOI:** 10.3389/fncel.2014.00349

**Published:** 2014-10-28

**Authors:** Mohamed Ouzzine, Sandrine Gulberti, Nick Ramalanjaona, Jacques Magdalou, Sylvie Fournel-Gigleux

**Affiliations:** UMR 7365 CNRS-Université de Lorraine “Ingénierie Moléculaire, Physiopathologie Articulaire”Vandoeuvre-lès-Nancy, France

**Keywords:** UDP-glucuronosyltransferases, blood-brain barrier, drug glucuronidation, detoxication barrier, glucuronidation of endogenous compounds

## Abstract

UDP-glucuronosyltransferases (UGTs) form a multigenic family of membrane-bound enzymes expressed in various tissues, including brain. They catalyze the formation of β-D-glucuronides from structurally unrelated substances (drugs, other xenobiotics, as well as endogenous compounds) by the linkage of glucuronic acid from the high energy donor, UDP-α-D-glucuronic acid. In brain, UGTs actively participate to the overall protection of the tissue against the intrusion of potentially harmful lipophilic substances that are metabolized as hydrophilic glucuronides. These metabolites are generally inactive, except for important pharmacologically glucuronides such as morphine-6-glucuronide. UGTs are mainly expressed in endothelial cells and astrocytes of the blood brain barrier (BBB). They are also associated to brain interfaces devoid of BBB, such as circumventricular organ, pineal gland, pituitary gland and neuro-olfactory tissues. Beside their key-role as a detoxication barrier, UGTs play a role in the steady-state of endogenous compounds, like steroids or dopamine (DA) that participate to the function of the brain. UGT isoforms of family 1A, 2A, 2B and 3A are expressed in brain tissues to various levels and are known to present distinct but overlapping substrate specificity. The importance of these enzyme species with regard to the formation of toxic, pharmacologically or physiologically relevant glucuronides in the brain will be discussed.

## The UDP-glucuronosyltransferase family

UDP-glucuronosyltransferases (UGTs, EC 2.4.1.17) are a multigenic family of enzymes responsible for the glucuronidation reaction, a main process of phase II xenobiotic biotransformation (Mackenzie et al., 2005). Products of phase I reactions mediated by P450-dependent monooxygenases are major substrates of UGTs, although substances which possess a functional chemical groups (hydroxyl-, phenyl, carboxylic acid, amines, thiols) are readily glucuronidated. Some of these substances especially hydroxylated or phenolic molecules can also be sulfated by sulfotransferases (SULTs). These enzymes compete with UGTs towards the same substrates. UGTs as monooxygenases, are membrane-bound enzymes. They are predominantly associated to the endoplasmic reticulum. If cytochromes P450 are expressed on the cytosolic surface of the membranes, the UGTs are present on the luminal side. Indeed intra-membrane co-localization and interactions of cytochromes P450 and UGTs insure a topological and functional coupling for an efficient stepwise drug biotransformation (Ouzzine et al., 1999; Ishii et al., 2007).

**Figure 1:**
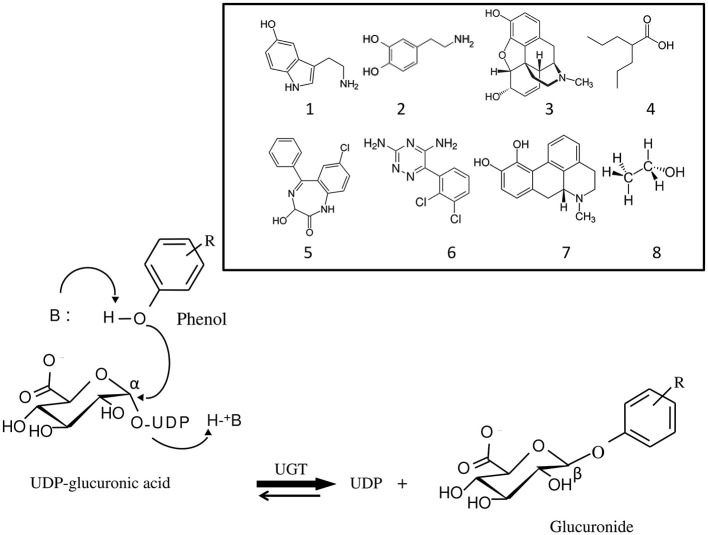
**Mechanism of the glucuronidation reaction catalyzed by UDP-glucuronosyltransferases (UGT)**. Glucuronidation is a bi-substrate reaction which requires an aglycone (for example, a phenol) and a high energy glucuronic acid donor, UDP-α-D-glucuronic acid, which is the common substrate to all UGT isoforms. The reaction leads to the release of UDP and formation of a β-D-glucuronide. UGT belongs to the inverting glycosyltransferase family which utilizes a direct S_N_2-like mechanism involving a base (B) catalyst. Structurally unrelated substances, brain transmitters and drugs against brain disorders that are UGT substrates are shown: 1, serotonin; 2, dopamine; 3, morphine; 4, valproic acid; 5, oxazepam; 6, lamotrigine; 7, apomorphine; 8, ethanol.

UGTs are glycosyltransferases that catalyze the covalent binding of glucuronic acid from the high energy donor, UDP-αD-glucuronic acid on structurally related substances with a functionalized nucleophilic group, leading to the formation of water soluble β-glucuronides and UDP (Figure 1). The reaction is believed to occur according to a second order nucleophilic substitution (S_N_2) involving an amino acid base catalyst (Magdalou et al., 2010). The main characteristic of UGTs is their potency to glucuronidate a large array of structurally unrelated substances. They play a major role in the detoxication of xenobiotics, including drugs and environmental substances, as well as in the metabolism of endogenous compounds (bilirubin, steroid hormones, bile acids, fatty acids) (Rowland et al., 2013). As such, they efficiently contribute to the protection of the organism against hazardous chemicals, and regulate the activity of several endogenous mediators involved in cell growth and differentiation. Many therapeutic classes of drugs, some of them targeting brain tissues, such as analgesics, anticonvulsants or antipsychotics are UGT substrates (de Leon, 2003). UGTs are also involved in the glucuronidation of endogenous compounds. Particularly, the monoamine neurotransmitters, dopamine (DA) and serotonin (5-hydroxytryptamine, 5-HT) are substrates of UGTs which may play a regulating role in their physiological function and implication in brain disorders (Suominen et al., 2013).

This powerful detoxication barrier is due to the expression in hepatic and extrahepatic tissues of several isoforms exhibiting distinct but overlapping substrate specificity. In human up to 22 UGT isoforms have been identified to date belonging to 1A, 2A, 2B, 3A and 8A subfamilies, based on a 29 amino acids conserved signature involved in the binding site for the common substrate, UDP-glucuronic acid (Rowland et al., 2013). Among these families, UGT1A, 2A and 2B are particularly active in xenobiotic biotransformation. Interestingly, the UGT isoforms exhibit tissue specificity in terms of isoform present and level of expression. If liver contains the greatest amount and variety of UGT isoforms, other organs, such as kidney or the intestinal tract are known to express several isoforms in significant amount (Court et al., 2012; Harbourt et al., 2012).

UGT has also been found in brain and associated tissues. A glucuronidation activity has been reported upon incubation of microsomes from total brain of rat with xenobiotics and endogenous compounds known to be substrates of the enzyme (Suleman et al., 1993). This activity was much lower than that found in liver or in other extra-hepatic organs. These enzymes were progressively characterized at a protein and gene levels using immunohistochemistry (Martinasevic et al., 1998) and PCR techniques (Suleman et al., 1998), thus allowing a precise identification of the isoforms and their localization within the brain tissues.

In the brain, despite their low level of expression, UGTs participate, in cooperation with the other drug metabolizing enzymes and the various ABC efflux transporters, to a metabolic barrier which prevents efficiently the organ from the intrusion of xenobiotics. Particularly, the blood brain barrier (BBB) and the associated cells, such as the endothelial cells, play a critical role on the cerebral homeostasis. The nasal cavity which affords a direct route of entry for xenobiotics to the brain contains specific UGTs isoform that are highly expressed in the olfactory tissue. In this condition, variations of UGT expression by genetic (polymorphism), environmental (induction or repression), pathophysiological factors or during ontogenesis and perinatal development will affect the permeability and the metabolic function of this barrier prone to potential toxic effects.

This review is focused on the importance of UGT in brain function. Although if, in terms of drug metabolism, the relatively small level of expression of these enzymes in the brain areas would have a minor impact on the overall glucuronidation of xenobiotics, it surely can have a major effect when protecting locally a specific cerebral cell from the neurotoxicity of substances which enter the brain. In terms of brain homeostasis, UGTs play a subtle role in the fine tuning of the concentration of endogenous neurotransmitters, ligands of receptors, or in the modulation of the pharmacological effects of brain-directed drugs. The presence of brain specific UGT isoforms highlights the importance of this family of enzymes in this process.

## A limited number of UGTs are expressed in the blood-brain barrier

The drug transporters and drug metabolizing enzymes that are present in the BBB prevent access to the brain of some lipid-soluble drugs and potentially toxic substances. It has been shown, several years ago, that rat brain microsomes exhibit high glucuronidation activity towards the reference substrate, 1-naphthol (Ghersi-Egea et al., 1987). This glucuronidation activity was found to be rather low in human brain. Interestingly, the 1-naphthol activity was also present in rat brain microvessels (Ghersi-Egea et al., 1988), indicating the capacity of endothelial cells to metabolize this compound and to participate to the protection of the brain toward this substance and related toxic compounds. The 1-naphthol activity was attributed to the UGT isoform 1A6 (UGT1A6) as it has been demonstrated that other phenolic substrates conjugated by this isoform were also metabolized in brain (Suleman et al., 1993). At cellular level, the 1-naphthol UGT1A6 activity was 10-fold higher in astrocytes, as compared with neurons and endothelial cerebrovascular cells (Suleman et al., 1998). Investigation of the expression of UGT1A6 in brain has been carried out by several authors (Martinasevic et al., 1998; Suleman et al., 1998). The expression of UGT1A6 was detected in rat astrocytes, neurone homogenates and brain microsomes by immunoblotting and by reverse transcriptase-polymerase chain reaction (RT-PCR). The presence of UGT1A6 in neuronal cells, especially the pyramidal cells of the cortex and the granular cells in the cerebellum was further confirmed by immunohistochemical localization of UGT1A6 using specific antibodies (Martinasevic et al., 1998). However, this study did not support the presence of UGT1A6 in the microvasculature of the rat brain as previously suggested. Similarly, investigation of the expression of UGTs in human brain microvessels did not reveal the presence of UGT1A6 (Shawahna et al., 2011), suggesting that this isoform was not expressed or very weakly expressed in human brain microvessels compared to rat. In line with this, it has been shown that UGT-mediated metabolism of 1-naphthol was less prominent in human brain compared to rat brain, suggesting that species differences may exist (Ghersi-Egea et al., 1993).

In addition to UGT1A6, the presence of UGT1A7, an isoform known to glucuronidate benzo(a)pyrene hydroxylated metabolites was detected by RT-PCR in rat astrocytes (Gradinaru et al., 2012). As UGT1A6, the mRNA expression of this UGT isoform is inducible by xenobiotics (Kobayashi et al., 1998). Among UGT1A isoforms, UGT1A1 was shown to be expressed in rat cerebellum at the mRNA level (Shelby et al., 2003). However glucuronidation activity towards bilirubin, the main substrate of this UGT isoform, has not been detected in rat brain, thus suggesting that UGT1A1 was not present (Suleman et al., 1993).

Recently, the expression of UGT1A4 has been investigated in brain. This isoform is known to catalyze the N-glucuronidation of primary, secondary and tertiary amines, among those are anticonvulsivants, tricyclic antidepressants and antipsychotics, such as lamotrigine, doxepin or clozapine (Li et al., 2007; Kerdpin et al., 2009). Immunobloting and glucuronidation activity using lamotrigine as a substrate indicated the presence of this UGT isoform in human brain microvascular endothelial cells (Ghosh et al., 2013). Analysis of the expression of UGT1A4 by immunohistochemistry in brain from patients with epilepsy indicated high expression in BBB endothelial cells and neurons with variable levels of UGT1A4 expression among the brain specimens analyzed. Endothelial cells from brain microvessels of these patients showed high lamotrigine glucuronidation activity compared to cells from non-pathological brain (Ghosh et al., 2013), suggesting a role in the drug-resistant epileptic brain. Although preliminary, these results emphasize the potential importance of the UGT1A4 isoform in the biotransformation of psychiatric drugs in brain.

**Figure 2:**
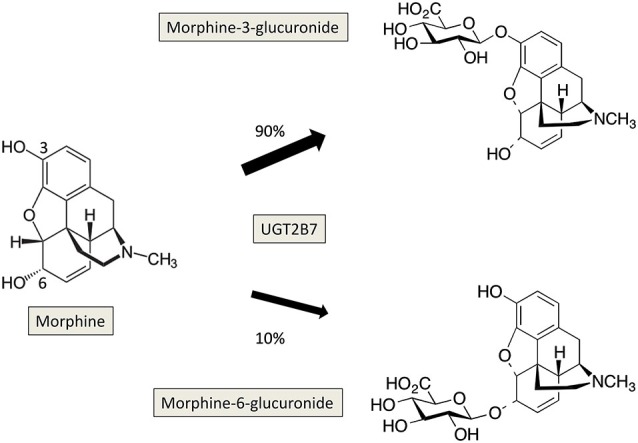
**Glucuronidation of morphine by UGT2B7 in human**. The reaction occurs on the two hydroxyl groups at positions 3 and 6 of morphine, and leads to the formation of 3-, and 6-glucuronides which present different pharmacological properties.

On the other hand, the UGT2B7 isoform catalyzes the glucuronidation of morphine to 3-O- and 6-O-glucuronide, the latter metabolite presenting a higher analgesic potency than morphine (Gong et al., 1991; Figure 2). It has been shown that morphine-6-O-glucuronide exhibited a slow transport across the BBB compared to morphine (Bouw et al., 2001), therefore the presence of UGT2B7 in brain may lead to local formation of morphine-6-O-glucuronide that can exert its analgesic actions. Investigation of the expression of UGT2B7 and of the formation of morphine glucuronides has been conducted in rat and human brain. In rat brain, analysis of the glucuronidation activity towards morphine indicated the absence of formation of morphine glucuronides (Suleman et al., 1993), suggesting that UGT2B7 may not be expressed in rat brain at least at basal levels. In contrast, the expression of UGT2B7 has been revealed by RT-PCR in human cerebellum 1, whereas no expression of this isoform was detected in brain cortex (King et al., 1999). Analysis of the glucuronidation activity of microsomes from human brain cortex towards morphine did not show any detectable activity confirming the absence of UGT2B7 in brain cortex. Only a weak activity was present in cerebellum 1. Noteworthy, Wahlström et al. (1988) reported the presence of morphine glucuronidation activity in human brain microsomes. However, this activity was detectable in only 3 out of 19 brain samples analyzed, suggesting large inter-individual variations in brain expression of this isoform. A recent model of transgenic mouse expressing human UGT2B7 has been developed showing a differential expression and activity of the enzyme in several tissues including brain to a lesser extent (Yueh et al., 2011). This animal model is a powerful tool to decipher the role of UGT2B7 *in vivo* and to understand the regulation mechanisms of its expression and activity under various experimental conditions (in presence of various biologically active compounds like drugs, toxics, hormones or in response to potential UGT inhibitors).

Interestingly, Christen and Fent (2014) recently reported the transcription profile of eight UGT genes in zebrafish tissues (*Ugt1a, Ugt1b, Ugt5a1, Ugt5a3, Ugt5a4, Ugt5a5, Ugt5c2, Ugt5c3*). They found they were significantly expressed in brain, especially the UGT5a and 5c families. However their catalytic function and implication in xenobiotic metabolism are not known yet. On the other, in the antennae of the pest insect, Spodoptera littoralis, Bozzolan et al. (2014) have characterized the expression of eleven putative UGTs that were differently regulated by odorants. Moreover, the isoform UGT46A6 was up-regulated by the insecticide deltamethrin. These data suggest a protective role of this enzyme in this olfactory organ towards xenobiotics.

Altogether, among the different UGT isoforms characterized so far in various tissues, it appears that only few of them are expressed in mammal and human brain. The construction of an anatomically comprehensive atlas of the adult human brain and mouse transcriptome which is currently under way will undoubtedly bring additional information on the presence of other or novel UGT isoforms (Lein et al., 2007; Sunkin and Hohmann, 2007).

## Role of glucuronidation in the metabolism of neurotransmitters

### Dopamine and serotonin conjugation in brain

DA and serotonin (5-hydroxytryptamine, 5-HT) are monoamine neurotransmitters. Their homeostasis in central and peripheral nervous system is important for many physiological processes as well as in pathological situations (Meiser et al., 2013). The function of DA has been linked to the regulation of motoric movements. The age-related Parkinson’s disease that coincides with the degeneration of dopaminergic neurons within the *substantia nigra* is associated with typical motor symptoms such as rigidity, tremor or bradykinesia. The discovery that Parkinson’s disease was associated with neostrial DA depletion (Ehringer and Hornykiewicz, 1960) led to the first treatment with L-3,4-dihydrophenelylalanine (DOPA, levodopa), which is still in use today. Schizophrenia and depression are other diseases linked to DA deregulation (Heinz and Schlagenhauf, 2010), and attention deficit hyperactivity disorder (ADHD) has been recently connected to deficient DA signaling (Tripp and Wickens, 2012). 5-HT is involved in many physiological functions such as body temperature regulation, blood pressure, perception of pain, sleep-wake cycles, and pathological processes including depression and anxiety (Berger et al., 2009).

**Figure 3:**
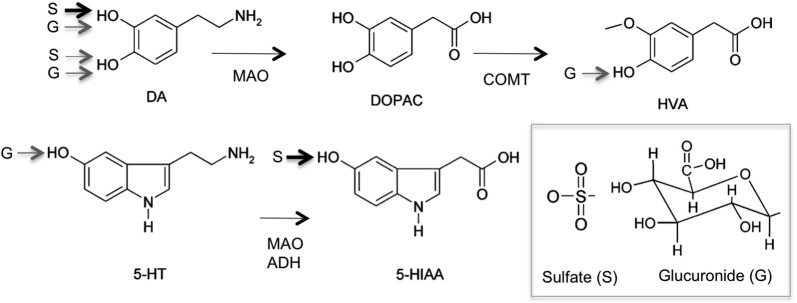
**Major metabolic pathway of serotonin (5-hydroxytryptamine) and dopamine (DA) to Phase I and Phase II metabolites (sulfate, S; glucuronide, G)**. Monoamine oxidase (MAO), aldehyde dehydrogenase (ADH) 3,4-dihydrophenylacetic acid (DOPAC), homovanillic acid (HVA), catechol-O-methyltransferases (COMT), 5-hydroxyindoleacetic acid (5-HIAA). Main sulfo- and glucuronoconjugate found in human brain are indicated by arrows (data from Suominen et al., 2013).

Upon neuronal excitation, DA is released into the synaptic cleft for signal transduction. DA signaling stops by reimport to the synaptic neuron and recycling, or degradation following uptake by glial cells. DA is primarily metabolized by oxidative deamination by monoamine oxidase (MAO) and aldehyde dehydrogenase-catalyzed reactions to 3,4-dihydrophenylacetic acid (DOPAC; Figure 3). DOPAC can be further metabolized to homovanillic acid (HVA) by catechol-O-methyltransferases (COMT). 5-HT is metabolized by MAO and aldehyde dehydrogenase to 5-hydroxyindoleacetic acid (5-HIAA; Figure 3). Both DA and 5HT and their respective metabolites can undergo conjugation with glucuronic acid or sulfonate mediated by UGTs and SULTs respectively, that occurs in both central nervous system and periphery. In the brain, phase I metabolites of DA i.e., DPAC and HVA, which are linked to the functional activity of dopaminergic neurons are predominant. However, in rat, mouse and human cerebrospinal fluid (CSF) that is assumed to reflect the metabolism of neurotransmitters, DA-glucuronide and sulfate conjugates have been found (Wang et al., 1983; Tyce et al., 1986; Uutela et al., 2009a). Due to the lack of commercially available standards, most conjugates in human, and animal CSF or brain samples have been analyzed after acid or enzymatic hydrolysis (Swahn and Wiesel, 1976). In addition, several studies report the detection of conjugates but do not specify the type of conjugate (Gordon et al., 1976; Tyce et al., 1985) leading to ambiguous results. However, in rat CSF, DA glucuronide was found predominant over DA-sulfate and free DA, suggesting that glucuronidation was an important metabolic pathway for DA of central origin (Wang et al., 1983). DA-glucuronide was also detected in human CSF samples following β-glucuronidase analysis (Tyce et al., 1986). The presence of intact glucuronide as the major DA conjugate was recently confirmed in rat and mouse brain microdialysates using liquid chromatography tandem mass spectrometry (LC-MS/MS; Uutela et al., 2009a). LC-MS/MS also detected 5-HT-glucuronides at concentration 2-times than HT itself (Uutela et al., 2009b). Altogether, the results indicated that in rat, neurotransmitters are glucuronidated whereas their phase I metabolites are sulfated (Uutela et al., 2009b). In human, early studies identified sulfate conjugates of DA and 5-HT (Ratge et al., 1985; Tyce et al., 1986) and glucuronide conjugate of DA in CSF. Glucuronide conjugates of HVA, DOPAC and 5-HIAA and DOPAC sulfate were detected in a caudate nucleus human sample although HVA and 5-HIAA occurred predominantly as free metabolites (Swahn and Wiesel, 1976). Intact 5-HT and HVA-glucuronides were detected in human brain samples. However, no glucuronides were detected in CSF samples. These recent results using a direct UPLC-MS-MS method clearly indicate that sulfate conjugation of neurotransmitters predominates over glucuronidation in the human brain (Suominen et al., 2013).

### UGT isoforms involved in dopamine and serotonin glucuronidation

Among 22 recombinant human UGTs, only UGT1A10 was found to catalyze DA glucuronidation at substantial rate although with a low affinity, leading to the formation of both 4-O- and 3-O-glucuronide (Itäaho et al., 2009). Very low activity was detected for UGTs 1A6, 2A1, 2A3, 2B7, 2B11 and 2B17. However, no expression could be detected in human brain that could be responsible for DA-glucuronide formation (King et al., 1999; Itäaho et al., 2009). UGT1A10 was also the most active human UGT in the formation of HVA-O-glucuronide whereas UG2A1 conjugated HVA at the carboxyl-group (Suominen et al., 2013). 5-HT has been shown to be a substrate for UGT1A6 and was proposed as a probe for this isoform (King et al., 1999). However, we were not able to detect 5-HT glucuronidation in recombinant cells expressing UGT1A6 in our laboratory (Fournel-Gigleux et al., 1991). This may be due to the low efficiency of UGT1A6 towards its substrate compared to the reference compound, 1-naphtol (8-fold). UGT2B7 also exhibited very low activity towards this substrate (King et al., 1999). The recombinant mouse Ugt1a6a and Ugt1a6b isoforms were able to glucuronidate 5-HT with approximately same efficiencies (Uchihashi et al., 2013). Since Ugt1a6a was predominantly expressed in mouse brain, especially in the hippocampus, it was proposed that this isoform is involved in the glucuronidation 5-HT in mouse brain. Altogether, monoamine neurotransmitters and their metabolites appear as poor substrates for recombinant human UGTs.

### Physiological significance of neurotransmitters glucuronidation in brain

The main excretion products of DA found in human urine are HVA, DOPAC, their sulfates and glucuronides, as well as DA conjugates. In the brain, DA and 5-HT conjugation seems to play only minor roles as in microdialysates, the main metabolites are DOPAC and HVA for DA, and HIAA for 5-HT. Whether glucuronidation is more or less important than sulfoconjugation is also questionable. Reports concerning the ratio of conjugated to non-conjugated metabolites and the ratio of sulfates to glucuronides are variable. In mouse and rat, there are mainly glucuronides over sulfates, for example, the concentration of 5-HT-glucuronide was about 2.5-times higher than of free 5-HT in rat brain microdialysates (Uutela et al., 2009b). On the other hand, in human CSF, 5-HIAA-sulfate and DA-3-O-sulfate were the predominant conjugates of 5-HIAA and DA respectively, no glucuronides were detected (Suominen et al., 2013). Quantitative results of DA and 5-HT metabolism indicate that sulfation is a more important phase II metabolism in human brain. Overall, the low glucuronidation activities towards DA and 5-HT, together with the UGT expression level found in brain tissues, and the poor efficiency of recombinant UGTs are indicative of a minor role of glucuronidation in DA and serotonin metabolism. The role of neurotransmitters conjugates in the brain remains to be clarified. Whether they exert neurotoxic or neuroprotective properties or have pharmacological impact on brain function, especially in the case of sulfate conjugates is an interesting question that requires further investigation.

## Role of UGT in the glucuronidation of xenobiotics in brain

As indicated above, several lines of evidence highlight the important role of UGT in brain homeostasis and neuronal protection against the entry of drugs and other xenobiotics. In order to illustrate this part, the glucuronidation of several xenobiotics in brain is reported.

### Morphine glucuronidation in brain

Morphine is one of the most potent and widely used opioid derivatives for acute or chronic pain relief. Once absorbed and distributed in blood flow, morphine is efficiently transferred across the BBB of rodent brain (Kalvass et al., 2007; Boström et al., 2008). The diffusion process varies according to the age or to the injected morphine dose (Bhargava et al., 1993). Interestingly, morphine can also be produced *in situ* in several tissues, including brain, from endogenous precursors such as tyramine and DA, (Laux-Biehlmann et al., 2013). *De novo* morphine synthesis from DA or L-tyrosine has been described in mammalian brain (Stefano et al., 2008) or human neuronal catecholamine-producing cell line SH-SY5Y (Poeaknapo, 2005; Muller et al., 2008). This biosynthetic pathway has been investigated by the use of potential precursors or deficient animal models such as DA-deficient mice. The results showed the requirement of DA for morphine formation (Neri et al., 2008). It has been strongly suggested that the presence of endogenous morphine in several tissues could be involved in pain modulation in response to various stimulations as stress, immune responses (Charron et al., 2011) or sepsis (Glattard et al., 2010). The recent (co)localization of morphine and morphine-like compounds in mouse brain areas not usually known to be involved in brain responses to pain brings new questionings about the potential other(s) role(s) of morphine and its derivatives (beside antalgic activity) in mammalian brain (Laux et al., 2011).

Morphine is mostly metabolized by glucuronidation leading to the production of two metabolites: the pharmacologically inactive and major metabolite, morphine-3-β-D-glucuronide (M3G) and the highly active but minor metabolite, morphine-6- β-D glucuronide (M6G; Figure 2; Nagano et al., 2000; De Gregori et al., 2012). A recent work reported the formation of M3G and M6G in primary cultures of neonatal rat microglia in presence of micromolar concentrations of morphine (Togna et al., 2013). The authors suggested that morphine glucuronides found in the CSF after morphine administration was in part formed in situ. As indicated before, among the UGT isoforms expressed in brain, UGT2B7 has been reported to glucuronidate morphine in several animal species and in humans (Court et al., 2003; Stone et al., 2003; Wong et al., 2006; Abildskov et al., 2010; Figure 2). Although UGT2B7 supported metabolism in liver is well described, the contribution of this UGT isoform in brain is still poorly documented and remains to be investigated in terms of pharmacological effects and potential consequences on brain homeostasis.

### Resveratrol glucuronidation in brain

Resveratrol (*trans*-3, 5, 4′-trihydroxystilbene) is a natural polyphenol produced by plants more particularly present in grapes and in high concentration in red wine. This compound has been regarded as a bioactive agent with possible beneficial effects in health. Its anti-oxidant properties may have therapeutic applications for cardiovascular or brain diseases. It has also been suggested that resveratrol could maintain neuronal energy homeostasis in relation to glutamate receptors and/or ions channels (Quincozes-Santos et al., 2014). Its neuroprotective effects have been shown in ischemia prevention in brain after a brief resveratrol pretreatment (Raval et al., 2008). Dasgupta and Milbrandt (2007) showed that resveratrol could activate the AMP-activated kinase (AMPK) in mouse neuroblastoma cell lines and in rat primary cultures of neurons, promoting neurite growth. These data strongly suggest that the neuroprotective effects of resveratrol could be mediated by the activation of AMPK in neurons (Dasgupta and Milbrandt, 2007).

Bioavailability of resveratrol is limited by its extensive biotransformation via sulfo- and glucuronidation pathways in rodents and humans (Iwuchukwu et al., 2012). This metabolism mainly occurs in kidney and in liver, and is low in rat brain (Juan et al., 2010). Resveratrol biotransformation leads to the production of various monoconjugated metabolites as 3- and 4′ monosulfates and 3- and 4′-monoglucuronides (Aumont et al., 2001; Sharan et al., 2012) that are excreted in urine. Previous studies showed that resveratrol was mainly metabolized by UGTs in rat brain homogenates, astrocyte primary cultures and olfactory mucosa (OM). Interestingly, only resveratrol 3-O-glucuronide has been produced in these cellular extracts whereas neither resveratrol 4-O′-glucuronide, nor sulfated conjugates could be detected in the same experimental conditions (Sabolovic et al., 2007). However the UGT isoforms involved in these reactions have not been identified, even if UGT1A6 and UGT2B7 are known to glucuronidate resveratrol. Finally, using glioblastoma cell lines from rat and human, Sun et al. (2013) showed that mainly glucuronides were formed in rat, whereas sulfate metabolites were produced in human cellular extract.

### Ethanol glucuronidation in brain

Ethanol is primarily oxidized in the liver and also in BBB. Glucuronidation has been recently described as a novel ethanol biotransformation pathway, with the characterization of an ethylglucuronide metabolite (Walsham and Sherwood, 2012). This metabolite represents 0.02–0.06% of the ethanol intake (Janda et al., 2002). Several recombinant UGT isoforms including UGT1A6 and UGT2B7 have been shown to catalyze the formation of ethylglucuronide (Schwab and Skopp, 2014). The ethylglucuronide presents a much longer half-life than ethanol itself and its relevance in clinical diagnostics has been suggested as a marker of alcohol consumption (Rainio et al., 2014). In line, previous studies have measured high concentrations of ethylglucuronide in CSF and brain of patients who died of acute alcohol intoxication (Wurst et al., 1999). The molecular mechanism for ethylglucuronide action has not been precisely described but it has been recently suggested that it could induce the toll-like receptor 4 signaling pathway leading to pain enhancement (Lewis et al., 2013). Ethylglucuronide formation exemplifies the biological importance of glucuronidation in brain. The mechanism of formation and action requires further investigation.

### Polychlorinated biphenyl glucuronidation in brain

Brain can also be a potential target for environmental pollutants. Polychlorinated biphenyls (PCB), which are highly toxic molecules present in persistent organic pollutants such as fumes and cigarette smoke have been described to be the cause of neurological adverse effects for years. A recent work showed a delay in the neuronal migration in fetal cortex following a prenatal exposure of a PCB mixture (Naveau et al., 2014). However, the mechanism underlying PCB toxicity in brain has not been clearly described yet. It has been hypothesized that the PCB neuronal deleterious effects could be related in part with the formation of one of their phase I metabolites, the hydroxyl-PCB produced by cytochrome P450 monooxygenases (Fonnum and Mariussen, 2009). This metabolite is glucuronidated *in vitro* by the rat UGT1A6 isoform which is expressed in brain (Daidoji et al., 2005). Taken together, these results strongly suggest that this UGT could be involved in hydroxyl-PCB glucuronidation in brain which could represent a PCB detoxification pathway and prevent its neurotoxicity.

In conclusion to this part, brain tissue is able to glucuronidate various xenobiotics which can cross the BBB. This reaction mainly leads to the neutralization of their toxicity or pharmacological activity. However, the potency of the brain to hydrolyze the glucuronides once formed has not been extensively explored. This reaction which is catalyzed by β-glucuronidases, leads back to the parent compounds. The balance glucuronidation/hydrolysis reaction must be taken into account to estimate the actual concentration of xenobiotics in the brain.

## The brain toxicity of bilirubin is associated to a defect in bilirubin glucuronidation in the liver

**Figure 4:**
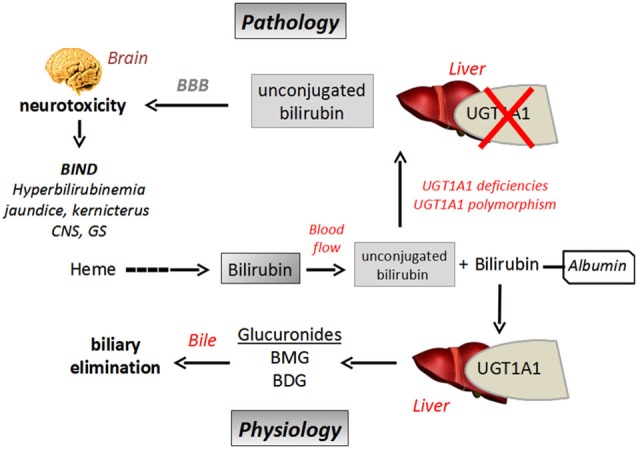
**Schematic representation of bilirubin biliary elimination pathway and neurotoxicity in a context of UGT1A1 defect**. BBB, Blood Brain Barrier; BIND, Bilirubin Induced Neurological Dysfunction; BMG, bilirubin monoglucuronides; BDG, bilirubin diglucuronide; UGT, UDP-glucuronosyltransferase.

Bilirubin is a toxic product of heme catabolism of hemoproteins produced in high amount. Normal plasma concentration of bilirubin has been measured between 2–10 mg/l in healthy people, which results from a daily production of about 200–300 mg of bilirubin. Once produced, the main excretion/detoxication pathway of the pigment is through glucuronidation supported by the UGT1A1 isoform (Ohta et al., 2005). The reaction leads to the production of bilirubin monoglucuronides (BMG) and bilirubin diglucuronide (BDG) which are finally excreted into bile and feces (Figure 4).

Indeed, when bilirubin is less metabolized, because of an immature glucuronidation capacity, as in newborns for instance (neonatal icterus), or in relation to UGT polymorphism (*see below*), unbound (free) plasma bilirubin concentration increases. The hydrophobicity of bilirubin explains its ability to efficiently permeate the BBB by passive transport (diffusion), resulting in its accumulation in brain where the molecule exerts its neurotoxic effects (Gazzin et al., 2012; Figure 4). In this context, glucuronidation of bilirubin in tissues could be regarded as a regulation mechanism to control its diffusion through BBB, as only free/unconjugated bilirubin can reach the brain (McDonagh, 2010). It has also been shown that high free bilirubin concentrations disrupt the integrity of human brain microvascular endothelial cells in primary cultures or in monolayer cultured-cell lines in terms of permeability and secreting activity. It has been suggested that prolonged and high exposition to bilirubin could cause brain endothelial cell injury by an oxidative stress-mediated mechanism in relation to a loss in glutathione homeostasis, increase in nitrite oxide production and cytokine (IL6) release (Palmela et al., 2011).

UGT1A1 is the only UGT described to date able to glucuronidate bilirubin. Therefore glucuronidation represents a rate-limiting step for the metabolism and elimination of the pigment. UGT1A1-mediated bilirubin conjugation has never been detected in brain, meaning that the conjugation of bilirubin in the other tissues, especially in the liver can be considered as a metabolic barrier to prevent its diffusion into the brain and thus to control its neurotoxicity. Recent studies on transgenic mouse expressing the human UGT1A gene locus (encoding 9 UGT1A genes, including UGT1A1) have been conducted, showing that about 8–10% of humanized UGT1A mice died from CNS damages with neuroinflammation and reactive gliosis as a consequence of neonatal hyperbilirubinemia (Fujiwara et al., 2010). High serum bilirubin concentrations have been observed in these transgenic animals in relation to limited UGT1A expression. More precisely, a delayed UGT1A1 expression has been pointed out, which explains hyberbilirubinemia and all the associated pathological features including bilirubin-mediated neurotoxicity (Chen et al., 2012).

The UGT1A1 gene is prone to an intense genetic variability in the human population including mutations on the open reading frame or in the promoter (TATA box) region leading to a decrease or even to a complete loss of bilirubin glucuronidation. Various pathological disorders have been described, such as the frequent, although benign Gilbert’s syndrome (Strassburg et al., 2008), or in contrast the rare but severe Crigler-Najjar syndromes (Bartlett and Gourley, 2011). All these diseases are characterized by moderate to high hyperbilirubinemia, respectively. A genetic defect in UGT1A1 and thus in bilirubin glucuronidation leads to high and potentially neurotoxic plasma bilirubin concentrations. When the concentration of the free fraction of the molecule exceeds the capacity of the UGT1A1 isoform, bilirubin becomes neurotoxic crossing the BBB, diffusing in specific brain regions (corpus striatum and hippocampus for instance) and inducing irreversible neuronal damages such as kernicterus (Brito et al., 2013).

In conclusion, bilirubin glucuronidation by UGT1A1 is a prerequisite and a key- step for detoxication of the pigment. Hepatic biotransformation of bilirubin by UGT1A1 controls its neurotoxicity by preventing its brain uptake and slowing down its BBB permeation, and finally by promoting its biliary excretion. On the other hand, controlling bilirubin amount in blood flow by increasing UGT1A1 expression seems to be an important strategy to prevent its neurotoxicity.

## UGT and olfactory tissues

In the previous sections, the important role of BBB and brain UGTs in the context of brain protection against xenobiotics has been discussed. However, since the last two decades, the olfactory system (OS) has emerged as an alternate barrier structure preventing penetration of inhaled toxic molecules into the brain. This assertion mainly relies on the early identification of some tissue-specific UGTs (Lazard et al., 1990, 1991), and cytochromes P-450 enzyme subfamilies (Nef et al., 1989; Zupko et al., 1991), in bovine, rat, and also human olfactory neuro-epithelium (Jedlitschky et al., 1999; Sneitz et al., 2009; Court et al., 2012). Indeed, these enzymes, which were first predicted to play important roles in chemoreception and especially in odorant signal termination (Nef et al., 1989; Lazard et al., 1991), are now ascertained as specific nasal xenobiotic metabolizing enzymes (Thiebaud et al., 2010; Xie et al., 2013). Nowadays, UGTs, due to their potency to glucuronidate a wide range of unrelated chemicals compounds, are considered as the main enzymes that confer to OS its known role as a metabolic barrier against potentially toxic inhaled substances. In mammals, OS consists in a neurophysiologic system including the OM and the olfactory bulb (OB) whose structures respectively allow generation, processing and driving of afferent odorant signals to the olfactory cortex (Mori et al., 2006). Since both OM and OB are able to express UGTs (Leclerc et al., 2002), OS can be considered as a metabolic defense structure against toxic airborne substances, in which OM and OB respectively represent an early and a late barrier stage. In this section, we will describe the UGTs isoforms which have been clearly identified in OM and OB, and their activities and roles in terms of brain protection against airborne substances intrusion.

### The olfactory mucosa as an early line of defense against toxic inhaled substances

The expression pattern of UGTs in the OM tissue has been investigated. Attention was first focused on the UGT2A1 isoform previously called UGTolf since Lazard et al. (1991) demonstrated that it was almost exclusively expressed in the olfactory tissue. They also detected by *in situ* immunolocalization the presence of UGT2A1 in the Bowman’s glands and in the apical pole of the sustentacular cells on serial frozen sections of bovine olfactory epithelium (Lazard et al., 1991). This result was later confirmed by Jedlitschky et al. (1999) in the human olfactory epithelium. By *in situ* mRNA localization and quantitative RT-PCR assays on rat olfactory epithelium, Heydel et al. (2001) reported that UGT2A1 mRNA was detectable in Bowman’s glands and sustentacular cells, and also in olfactory sensory neurons. These data were confirmed by a proteomic approach by Mayer et al. (2008). However, at a lesser extent, others UGT isoforms were also shown to be expressed in OM. Thus, Leclerc et al. (2002) described the presence of UGT1A6 mRNA in rats OM, although this latter isoform was 400 to 4,000 times less expressed than UGT2A1. Furthermore, high-throughput analysis of more than 6,000 cDNA mouse sequences indicated the expression of UGT2A2, a splice variant of UGT2A1, in neonatal OM whose glucuronidation activity towards several different endo- and xenobiotic substrates was similar to that of UGT2A1 (Strausberg et al., 2002; Sneitz et al., 2009; Court et al., 2012).

UGT2A1 is, thus, widely expressed among the cells which constitute the OM tissue. However, the expression of the UGT1A6 isoforms also contributes to the glucuronidation activity observed in the OM tissue. What does this co-localization mean? A preliminary answer would reside in the difference and complementarity of substrate specificity of UGT2A1 compared to that of UGT1A6. In one hand, UGT2A1 catalyzes mainly the glucuronidation of most odorant compounds including numerous phenols derivatives, aliphatic and monoterpenoid compounds, but also accepts numerous steroids as endogenous substrates including testosterone, 5α-androstane-17β-ol-3-one or 5α-androstane-3α-17β-diol (Jedlitschky et al., 1999). On the other hand, UGT1A6, which is expressed in various tissues (Ouzzine et al., 1994; Münzel et al., 1996), including brain (King et al., 1999) is mainly involved in the glucuronidation of planar phenols (Tukey and Strassburg, 2000), acetaminophen (Bock et al., 1987), serotonine (Krishnaswamy et al., 2003, 2004), but also glucuronidates carcinogenic arylamines and aryl hydrocarbons (Gschaidmeier et al., 1995). Moreover, UGT1A6 was recently shown to present a significant affinity toward nonsteroidal anti-inflammatory drugs and salicylate derivatives (Soikkeli et al., 2011). As expected, the chemical structure of the substrates recognized by UGT2A1 differs from that for UGT1A6 suggesting that conjugation by both enzymes allows widening the spectra of inhaled molecules that could be glucuronidated. This is consistent with the role of OM as metabolic structure preventing penetration of potentially toxic inhaled substances inside the brain. However, this assertion should be considered in the light of the recent studies regarding the complex expression regulation of these enzymes. Indeed, the expression level of UGT2A1 mRNA was shown to be significantly higher than that of UGT1A6 mRNA in rat OM but not in rat OB, indicating that olfactory UGT expression was tissue-dependent (Leclerc et al., 2002). The same authors also demonstrated that UGT2A1 and UGT1A6 expression in rat OM were age-dependent. Furthermore, Buckley and Klaassen (2007) showed that, in mice, female-predominant expression of UGT2A1 was observed in OM, while male-predominant expression of UGT1A6 was rather observed in lung, suggesting that expression of these OM UGTs could also be gender-dependent (Buckley and Klaassen, 2007). Interestingly, Thiebaud et al. (2010) reported that UGT2A1 and UGT1A6 did not share the same gene transcription inducers in rat OM. In fact, UGT2A1 mRNA expression level was up-regulated by dexamethasone (DM) whereas no modulation effect of phenobarbital, Aroclor 1254, methylcholanthrene or ethoxyquin upon the residual UGT2A1 mRNA expression level was observed. Furthermore, these authors also showed that none of these compounds could up-regulate the UGT1A6 mRNA residual expression. Several xenobiotic-response transcription factors (XRTF) involved in the modulation of some UGTs expression in rat OM have been identified. These XRTFs, including aryl hydrocarbon receptor, nuclear factor E2-related factor 2, peroxisome proliferator-activated receptor, pregnane X receptor, and glucocorticoid receptor, are known to mediate UGTs mRNAs expression upon addition of specific inducers in rat OM (Thiebaud et al., 2010), but also in mouse liver and intestine (Buckley and Klaassen, 2009). Thus, such an approach would allow: (i) to decipher the molecular basis of the enzymatic response of OM towards inhaled substances (ii) to highlight transcription factors or metabolic partners which would be targeted in therapeutic aims (Rowland et al., 2013). Besides its known role as a neurosensory structure, OM can also be considered as a dynamic structure capable to adapt the expression of its xenobiotic metabolizing enzymes to terminate olfaction signal but also to eliminate toxic inhaled compounds in the context of brain protection.

### The olfactory bulb as a late line of defense against toxic inhaled substances

OB is a second line of defense of OS since this structure was also shown to express drug metabolizing enzymes, including UGTs (Leininger et al., 1991). OB main role is to process the olfactory signals received from olfactory sensory neurons to then ensure their transfer towards the olfactory cortex and towards other brain areas including the amygdala, the hippocampus, the piriform cortex, and the entorhinal cortex to mainly generate olfactory information and odor memory (Johnson et al., 2000; Buck, 2005; Mori et al., 2006; Zelano et al., 2009). Few groups attempted to identify the UGTs that were specifically expressed in OB tissue, and demonstrated the expression of the UGT2A1 and UGT1A6 isoforms. The expression was heterogeneously distributed among the different cells of OB. By *in situ* hybridization analysis, Heydel et al. (2001) successfully localized UGT2A1 mRNA expression inside the granule cells and in a lesser extent in some mitral cells whereas no signal was observed in the other layers of OB. Unfortunately, no such exploration was made for UGT1A6.

UGT1A6 was identified as the main enzyme responsible for the glucuronidation of 1-naphthol in rat OB (Gradinaru et al., 1999). The activity was age-dependent and a significant increase of UGT1A6 mRNA expression level was observed in OB for rats older than 3 months (Leclerc et al., 2002). Interestingly, UGT2A1 was expressed in rat OB, although at a less amount than in OM. mRNA expression level increased for rats from 1-day up to 3-months-old, then decreased thereafter (Leclerc et al., 2002). These data are important as they suggest that OB possesses the same enzymatic potency as OM against toxic inhaled substances. These data also suggest that, as a function of age, UGT1A6 likely plays a greater role in the glucuronidation activity when compared to UGT2A1. Interestingly measurement of glucuronidation activity toward a series of structurally unrelated hydroxylated substances including odorants and phenols suggested that other UGT isoforms may be expressed in rat OB. To better understand how OB exerts its detoxification role to ensure brain protection, further exploration regarding the identification of such hypothetical isoforms should be undertaken.

The results acquired during these last two decades undoubtedly indicates that, more than a complex signal processing structure, OB acts as an additional line of defense against toxic substances that would target the cerebral tissue thanks to the presence of UGT2A1 and UGT1A6 enzymes. Moreover, this substructure of the OS has to be considered as a “late” line of defense, because OB is already recognized as a part of the forebrain and thus belongs to the cerebral tissue.

## Conclusions

Compared to other organs, brain tissues are characterized by a qualitatively and quantitatively low expression of UGTs. Only few isoforms are present which are heterogeneously distributed among the several cells constituting the different brain areas. Beside their significant participation to the overall protection of the brain against the entry of xenobiotics, including pollutants present in our environment, these enzyme species are specialized toward the biotransformation of brain-directed substances, including metabolism of neurotransmitters chemically related to DA, or antipsychotic drugs. However, the contribution of UGTs expressed in liver or in gastro-intestinal tract in the protection of the brain against drugs and other xenobiotics has to be taken into account. By actively eliminating these substances from the body, they decrease the risk of their entry into the brain. On the other hand, a better knowledge on the UGTs, and other drug metabolizing enzymes and transporters in the BBB and olfactory tissues is a prerequisite to design and target pharmacological compounds toward brain able to bypass these physiological barriers.

## Conflict of interest statement

The authors declare that the research was conducted in the absence of any commercial or financial relationships that could be construed as a potential conflict of interest.

## Abbreviations

BBB: blood-brain barrier
CSF: cerebrospinal fluid
DA: dopamine
5-HT: 5-hydroxytryptamine
OB: olfactory bulb
OM: olfactory mucosa
OS: olfactory system
UGT: Uridine-diphosphate-glucuronosyltransferase.
